# Acute Nontraumatic Clavicle Fracture Associated with Long-Term Bisphosphonate Therapy

**DOI:** 10.1155/2014/986718

**Published:** 2014-08-31

**Authors:** Shen Hwa Vun, Yahya Husami, Sajan Shareef, Diane Bramley

**Affiliations:** ^1^Trauma and Orthopaedics Department, Monklands Hospital, Monkscourt Avenue, Airdrie, North Lanarkshire ML6 0JS, UK; ^2^Department of Radiology, Monklands Hospital, Monkscourt Avenue, Airdrie, North Lanarkshire ML6 0JS, UK

## Abstract

Cases of osteonecrosis of the jaw, insufficiency fractures and atypical low energy or atraumatic fractures of pelvis, femur (subtrochanteric/mid-shaft/distal-third), tibia, fibula, metatarsal, humerus, and ulna related to long-term bisphosphonate therapy have been reported in the literature. We present the case of an acute nontraumatic clavicle fracture, associated with long-term bisphosphonate therapy, which to our knowledge has not been reported previously. This case highlights the need of critical evaluation of patients with atypical fractures during long-term bisphosphonate therapy.

## 1. Introduction

Bisphosphonates have been widely used in both the treatment and prevention of osteoporosis and osteopenia. It is well known for its efficacy in decreasing bone resorption and hypercalcemia, reducing osteolysis of bone metastasis, and its safety profile [1]. Several randomized clinical trials have also shown that bisphosphonates increase bone density and reduce the risk of vertebral, nonvertebral, and hip fractures [2, 3].

However, it has been suggested recently that the prolonged suppression of bone turnover under long-term administration may impair the ability of bone to remodel, leading to the accumulation of microdamage, compromised bone strength [4, 5], and ultimately progression to insufficiency fracture. Cases of osteonecrosis of the jaw; insufficiency fractures and low energy or atraumatic fractures of the pelvis [6, 7]; femur involving the subtrochanteric, mid-shaft, and distal-third level [4, 8–13]; tibial diaphysis [14–16]; fibula [8]; metatarsal [8]; humerus [13]; and ulna [17] have been reported.

We report a case of an acute nontraumatic, transverse mid-clavicular fracture in a patient who has been on long-term alendronic acid.

## 2. Case Report

A 90-year-old postmenopausal woman presented to the accident and emergency (A&E) department with acute pain of her right shoulder after turning the tap off in her kitchen. She reported hearing a “crack” whilst turning the tap off and noticed an obvious bump over her right shoulder, which prompted her to attend the A&E department. Patient denied any history of previous trauma to her right clavicle, and there was no prodromal symptom such as pain over the right shoulder or clavicle.

**Figure 1:**
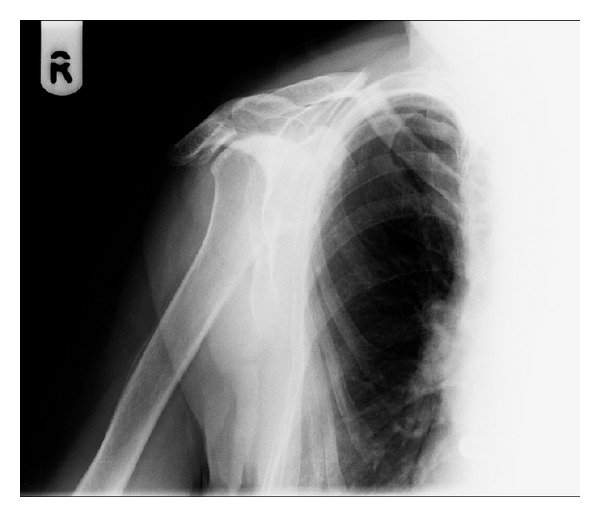
Radiograph taken in A&E department, demonstrating acute transverse right mid-clavicular fracture.

Radiographs revealed an acute transverse right mid-clavicular fracture, with evidence of superior cortical thickening and a small superior spike (Figure 1). Her only past medical history was osteoarthritis of her knees and bilateral varicose veins. She had been taking treatment dose of alendronic acid 70 mg once weekly for postmenopausal osteoporosis over the past seven years. Her other regular medication was Adcal-D3 tablets (1500 mg calcium carbonate and 400 iu colecalciferol) taken once daily and paracetamol 1 g tablets, taken four times a day. Her most recent serum 25 hydroxyvitamin D concentration was 140 nmol/L.

The patient was referred to our departmental fracture clinic a few days following her attendance in A&E. Apart from pain on movements of her right shoulder, patient's right upper limb was neurovascularly intact. There was no evidence of skin tenting or compromise on examination. She was therefore conservatively managed on a broad arm sling. Apart from the above-mentioned past medical history, she denied any signs and symptoms of systemic illnesses or malignancy. Blood tests including myeloma screen were all normal. Her initial radiographs (Figure 1) were suggestive of an atypical fracture associated with long-term bisphosphonate treatment; therefore, both patient and her general practitioner were advised to stop the regular alendronic acid prescription.

**Figure 2:**
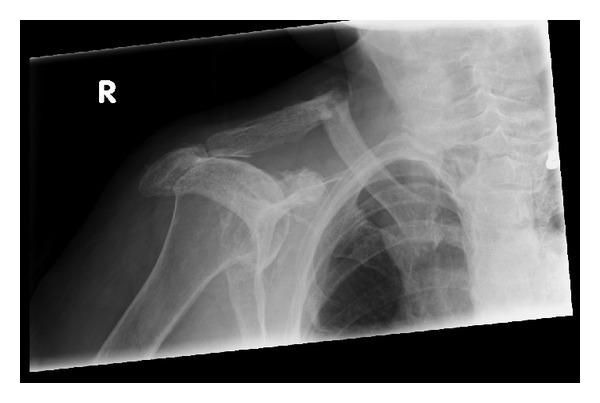
Follow-up radiograph at fracture clinic, four weeks following conservative management, demonstrating signs of callus formation of the right mid-clavicular transverse fracture. Superior cortical thickening and a small spike of the fracture fragments are also seen in this figure.

Follow-up radiographs 4 weeks later (Figure 2) demonstrated some signs of callus formation. Figure 2 clearly demonstrates the transverse fracture and cortical thickening of the superior cortex and a small spike. At four weeks of followup, clinically, there was no gross deformity over her right shoulder. The patient's pain had reduced and she was beginning to improve her range of movement. Following discussion with the patient, a decision was made to continue treating this fracture conservatively as it would not impact her normal activities.

## 3. Discussion

Bisphosphonates are potent inhibitors of bone resorption, often prescribed as a first-line therapy for postmenopausal osteoporosis. In comparison to all other pharmacological inhibitors of bone resorption, bisphosphonates are unique for their long-term retention in the bone and the persistence of their effect after therapy cessation. Bisphosphonates have a half-life of more than 10 years [18]. However, the prolonged suppression of bone turnover may impair the ability of bone to remodel, leading to accumulation of microdamage and compromised bone strength [4, 5].

Atypical fractures of the femur have been described in patients receiving alendronate for more than 5 years [10, 19]. Our patient had been taking alendronate therapy for 7 years. The incidence of these fractures was estimated to be approximately 78/100,000 in patients taking oral bisphosphonates [20]. As described by Giusti et al. [5], Odvina et al. [8], and Rizzoli et al. [21], these atypical fractures occur with minimal or no trauma.

Not only does this patient have no previous history of trauma, but also the fracture occurred at an atypical site (clavicle) during the simple mechanical action of turning off a tap. We have also excluded the possibility of malignancy and systemic illnesses. Furthermore, radiographs displayed appearances similar to those reported in the literature on atypical femoral fractures associated with long-term bisphosphonates treatment: simple transverse fracture in an area of thickened cortex (superior cortex of the clavicle in this case) with unicortical beaking [8–12]. The radiograph also demonstrated degeneration of acromioclavicular joint, osteoarthritis of the right glenohumeral joint, and secondary evidence of rotator cuff arthropathy (superior subluxation of humerus); all of these could have increased the stress on the clavicle, contributing to the fracture.

Although bisphosphonates were commonly identified as important risk factors for developing atypical fractures, it is worth noting that other factors such as medications (corticosteroids and proton-pump inhibitors), cortical geometry, and properties of bone matrix have been suggested as predisposing risk factors too [22–24]. Our case report supports the observations made by others, emphasizing the need for increased awareness of atypical/stress fractures during long-term bisphosphonate therapy.
